# New mechanistic insight into SARM1 activation: TRIM32-mediated ubiquitination is a key lever that actuates SARM1’s catalytic action, leading to axon degeneration and cell death

**DOI:** 10.3389/fncir.2026.1842408

**Published:** 2026-08-20

**Authors:** Hitoshi Murata, Nahoko Tomonobu, Ken-ichi Yamamoto, Rie Kinoshita, Masakiyo Sakaguchi

**Affiliations:** Department of Cell Biology, Okayama University Graduate School of Medicine, Dentistry and Pharmaceutical Sciences, Okayama, Japan

**Keywords:** axon degeneration, NAD ^+^, SARM1, TRIM32, ubiquitination

## Abstract

Sterile alpha and TIR motif-containing protein 1 (SARM1) is an enzyme that cleaves nicotinamide adenine dinucleotide (NAD^+^) and plays a role in disrupting neural circuits through axon degeneration and cell death. SARM1 is activated by changes in the nicotinamide mononucleotide (NMN)/NAD^+^ ratio and by various post-translational modifications, but its complete regulatory mechanism remains poorly understood. Here, we report that tripartite motif-containing protein 32 (TRIM32) activates SARM1 through specific ubiquitination triggered by anticancer drug treatment. TRIM32 promotes the attachment of Lys27-linked ubiquitin chains to Lys173 and Lys375 within the ARM domain, which is an autoinhibitory region of SARM1. This ubiquitination by TRIM32 increases SARM1’s NAD^+^-cleaving catalytic activity and enhances its ability to induce neurite degeneration and cell death. A mutant form of TRIM32 lacking the enzymatically active RING domain fails to promote SARM1 ubiquitination, and reducing TRIM32 levels decreases SARM1 ubiquitination. Additionally, ubiquitin-specific peptidase 13 (USP13), a known negative regulator of SARM1, can deubiquitinate SARM1. These findings suggest that TRIM32 is a key regulator of axon degeneration and cell death through its ubiquitination of SARM1.

## 1 Introduction

SARM1, a protein containing a sterile alpha and TIR motif, promotes axon degeneration and cell death through its NAD^+^-cleavage activity (Essuman et al., 2017; Gerdts et al., 2013; Summers et al., 2014; Figley and DiAntonio, 2020). Its pathological overactivation plays a crucial role in the progression of neurological diseases such as Alzheimer’s, Parkinson’s, amyotrophic lateral sclerosis, trauma, Charcot–Marie–Tooth disease, and chemotherapy-induced peripheral neuropathy (CIPN) (Miao et al., 2024; Peters et al., 2021; Veriepe et al., 2015; Marion et al., 2019; Sato-Yamada et al., 2022; Geisler et al., 2016; Turkiew et al., 2017). Since SARM1-knockout mice exhibit significantly fewer symptoms of these diseases (White et al., 2019; Cheng et al., 2019; Gilley et al., 2017; Gould et al., 2021; Henninger et al., 2016; Maynard et al., 2020), SARM1 has become a promising target for drug development. Several small molecules that inhibit SARM1’s NAD^+^-cleavage activity have been developed and shown to reduce symptoms by blocking SARM1 (Hughes et al., 2021; Bosanac et al., 2021; Bratkowski et al., 2022; Feldman et al., 2022; Khazma et al., 2022; Chen and Li, 2024). With the goal of suppressing SARM1, we also focused on plant-derived compounds that can serve as food nutrients, with the aim of ensuring safe use, and reported that carnosol, a compound from rosemary, alleviates CIPN symptoms through its SARM1-inhibitory activity (Murata et al., 2025).

SARM1 contains a mitochondrial targeting signal (MTS) that enables it to move to mitochondria, an armadillo/HEAT motif (ARM) domain with an autoinhibitory role, a tandem sterile alpha motif (SAM) domain involved in oligomerization, and a Toll/interleukin-1 receptor (TIR) domain with NAD^+^-cleavage activity. Typically, SARM1 remains inactive, with structural studies showing that the ARM and TIR domains interact to inhibit the NAD^+^-cleavage activity of the TIR domain (Bratkowski et al., 2020; Shi et al., 2022). Conversely, SARM1 becomes active and degrades NAD^+^ when exposed to stress from trauma or anticancer treatments, leading to axonal damage. It has been reported that the catalytic switch-ON compositional change affects the intracellular NMN/NAD^+^ ratio, leading to increased NMN binding to the ARM domain, which prompts the suppressor ARM domain to dissociate from the TIR domain, ultimately activating NAD^+^ degradation via the free and flexible TIR domain (Figley et al., 2021). In addition to the regulatory role of NAD^+^ imbalance in SARM1, several other mechanisms that control its activity have been reported. Wang et al. reported that double-stranded DNA binds to the SARM1 TIR domain, thereby triggering its catalytic activity (Wang et al., 2025). Shi et al. revealed another mechanism in which Caspase-3 cleaves the SARM1 ARM domain, thereby activating its NAD^+^-cleavage activity (Shi et al., 2026). We also found that c-Jun N-terminal kinase (JNK)-mediated phosphorylation of Ser548 in SARM1 increases its NAD^+^-cleavage activity (Murata et al., 2018). Phosphorylation of Ser548 promotes mitochondrial targeting of SARM1 and enhances axon degeneration (Xue et al., 2021). However, the full picture of the mechanisms regulating SARM1 activity remains incompletely understood. For example, it remains unclear how many regulatory mechanisms SARM1 has, whether they operate in a single manner suited to specific cellular stress conditions, whether they exhibit mutual or reciprocal patterns, and, if so, how they are ordered in driving SARM1 activation. To clarify these points, the first step is to discover and elucidate SARM1’s regulatory mechanisms one by one in detail.

Interestingly, Yue et al. recently reported that ubiquitin-specific peptidase 13 (USP13), a deubiquitinating enzyme, inhibits SARM1 activation and reduces axon degeneration (Yue et al., 2023). USP13 has been shown to interact with the ARM domain of SARM1 and deubiquitinate it. This suggests that SARM1 activation may also be regulated by ubiquitination. However, the enzyme responsible for SARM1 ubiquitination and the specific ubiquitination site on SARM1 have not yet been identified.

In this study, we aimed to clarify the mechanism of SARM1 ubiquitination. Our current efforts have identified the target, revealing that tripartite motif-containing protein 32 (TRIM32) is a potential E3 ubiquitin ligase for SARM1. TRIM32 promotes SARM1 ubiquitination by interacting with SARM1. It modifies SARM1’s structure by attaching ubiquitin chains to its ARM domain, which increases SARM1 activity. USP13 removes the ubiquitin chains added by TRIM32 and reduces TRIM32-mediated SARM1 activation. These findings could help develop preventive and therapeutic strategies targeting SARM1 to protect neural circuits from severe damage caused by neurological diseases.

## 2 Materials and methods

### 2.1 Chemicals and antibodies

Vincristine sulfate and Paclitaxel were purchased from FUJIFILM Wako Pure Chemical Corporation (Osaka, Japan). Nicotinamide riboside (NR) was purchased from Cayman Chemical (Ann Arbor, MI). Vacor (Pyrinuron) was obtained from Chem Service (West Chester, PA). The following antibodies were used: peroxidase-conjugated rat anti-HA (Merck; cat#12013819001), rabbit anti-FLAG (DDDDK-tag) (MBL, Nagoya, Japan; cat#PM020), mouse anti-Myc tag (Cell Signaling Technology, Beverly, MA [CST]; cat#2276), mouse anti-His tag (CST; cat#2366), rabbit anti-SARM1 (CST; cat#13022), rabbit anti-TRIM32 (Proteintech, Rosemont, IL; cat#10326-1-AP), and mouse anti-*β*-actin (Sigma-Aldrich, Tokyo; cat#A2228). The mouse monoclonal antibody (mAb) against phospho-Ser548 SARM1 was generated by ITM Co. (Matsumoto, Japan) (Murata et al., 2018). The rabbit polyclonal antibody against SARM1 for immunoprecipitation was produced by Sigma-Aldrich. This antibody was generated by immunizing animals with a synthetic peptide corresponding to residues surrounding the ARM domain of human SARM1 (WWAAGGRGPREVSPGAGTEV).

### 2.2 Plasmids

Conventional molecular biological techniques were employed to create the following expression constructs: C-terminal Flag-tagged human wild-type (WT) SARM1, a SARM1 mutant with the TIR domain deleted (ΔTIR, removing amino acids 551–724), and SARM1 mutants with point mutations (K80R, K173R, K363R, K375R, S548A, and 2KR [K173R and K375R]). Additionally, N-terminal HA-tagged human WT ubiquitin and ubiquitin mutants with point mutations (K8R, K11R, K27R, K29R, K33R, K48R, and K63R), along with C-terminal Myc-tagged or C-terminal 3Myc-6His tagged WT TRIM32 and TRIM32 mutants with domain deletions (ΔRING, deleting amino acids 18–62; ΔB-box, deleting amino acids 99–134; ΔCC, deleting amino acids 138–197; ΔNHL, deleting amino acids 371–643). The constructs also included N-terminal HA-tagged human WT JNK1, and C-terminal 6His-tagged human WT USP13 and USP13 mutants with a point mutation (C345A). These constructs were ligated into the pIDT-SMART (C-TSC) plasmid vector (pCMViR-TSC) (Sakaguchi et al., 2014). Point mutations and gene deletions were generated by inverse PCR using a KOD-Plus mutagenesis kit (Toyobo). All expression constructs were sequenced to confirm correct reading frames and ensure the absence of additional mutations.

### 2.3 Cell culture

HEK293T and SH-SY5Y cells were cultured in DMEM/F12 medium (Thermo Fisher Scientific, Waltham, MA) supplemented with 10% fetal bovine serum (FBS). To generate mature human neuronal cells in culture, 201B7-induced pluripotent stem cells (iPSCs) derived from a healthy 36-year-old female donor (RIKEN BRC, Tsukuba, Japan) were used and differentiated according to a previously established protocol (Murata et al., 2023). Neural stem cells (NSCs) from iPSCs were induced using PSC neural induction medium (Thermo Fisher Scientific) according to the manufacturer’s instructions. After 7 days of neural induction, P0 NSCs were expanded in neural expansion medium on dishes coated with Geltrex LDEV-Free hESC-qualified reduced growth factor basement membrane matrix (Thermo Fisher Scientific). To prepare the Geltrex-coated dish, it was incubated with Geltrex matrix solution (diluted 1:100 in Neurobasal medium) for 1 h. For neuronal differentiation, NSCs were cultured at a density of 1 × 10^5^ cells/cm^2^ on dishes coated with 0.002% poly-L-lysine (Sigma-Aldrich), followed by 10 μg/mL laminin (Thermo Fisher Scientific). The culture medium used was Neurobasal Plus (Thermo Fisher Scientific) supplemented with 2% B-27 Plus (Thermo Fisher Scientific), 2 mM GlutaMAX (Thermo Fisher Scientific), CultureOne (Thermo Fisher Scientific), and 200 μM ascorbic acid (Sigma-Aldrich). The medium was replaced every 3 days, and the cells were used for experiments after 6 days of culture. Cells within passages 5 to 20 were utilized in the experiments.

### 2.4 Cell transfection

For plasmid transfection, cells were transfected with the indicated plasmids using FuGENE-HD (Promega Biosciences) according to the manufacturer’s instructions. Transfection was performed at 40% cell density. For experiments using 6-well plates, 2 μg of plasmids and 4 μL of FuGENE-HD were used. For RNA interference, Stealth RNAi siRNA targeting TRIM32 (HSS177054 and HSS177055; Thermo Fisher Scientific) was transfected at 20 nM using Lipofectamine RNAiMAX (Thermo Fisher Scientific). Stealth RNAi siRNA negative control (Med GC; Thermo Fisher Scientific) served as a negative control.

### 2.5 The establishment of stable transformants of NSCs and differentiation to neurons

The pSAKA-1B plasmid vector (Sakaguchi et al., 2024) was used to generate stable transgenic NSC clones expressing the target genes. Human NSCs were transfected with the target plasmids and a plasmid encoding the GFP-puromycin composite resistance gene (GFP-2A-puro) using Lipofectamine Stem Transfection Reagent (Thermo Fisher Scientific). After 48 h, the transfected NSCs were treated with 0.5 μg/mL puromycin, and clones that stably expressed the foreign genes along with GFP-2A-puro were selected. These selected NSC clones were then differentiated into neurons following the same procedures described above.

### 2.6 Cell viability assay

A CellTiter-Glo assay (Promega Biosciences) was performed to assess cell viability. Cells were incubated with the CellTiter-Glo detection reagent for 10 min, following the manufacturer’s instructions. Luminescence was measured using a Fluoroskan Ascent FL plate reader (Thermo Fisher Scientific). Cell viability was expressed relative to the control group, which was set at 100%.

### 2.7 NAD^+^ assay

To analyze intracellular NAD^+^ levels, a Cell Count Normalization Kit (Dojindo, Kumamoto, Japan) was used to standardize cell numbers prior to the NAD^+^ assay. The fluorescence intensity of cells stained with Hoechst 33342 was measured using a FlexStation 3 microplate reader (Molecular Devices, Sunnyvale, CA; Ex: 350 nm; Em: 461 nm). An NAD/NADH-Glo assay (Promega Bioscience) was employed to determine NAD^+^ levels. Cells were incubated with the NAD/NADH-Glo reagent for 30 min following the manufacturer’s instructions. The luminescence intensity of cells treated with the NAD/NADH-Glo reagent was detected using a Fluoroskan Ascent FL plate reader (Thermo Fisher Scientific). NAD^+^ levels were normalized to the fluorescence of Hoechst 33342.

### 2.8 Immunoprecipitation

Cells were lysed in an ice-cold lysis buffer (40 mM Tris–HCl, pH 7.4, 150 mM NaCl, and 1% Triton X-100). After centrifugation, supernatants were incubated with 20 μL of a 50% slurry of anti-FLAG-M2 affinity gel (Sigma-Aldrich) or anti-Myc-tag mAb-agarose (MBL) for 3 h. To immunoprecipitate endogenous SARM1, supernatants were incubated with 10 μL of anti-SARM1 antibody (Sigma-Aldrich) and 20 μL of Dynabeads Protein G (Thermo Fisher Scientific) for 3 h. Immunoprecipitates were washed twice with the lysis buffer, eluted with 0.1 M Gly-HCl (pH 2.5) solution, and immediately neutralized with 1 M Tris–HCl (pH 9.0) and SDS sample buffer.

### 2.9 Western blot analysis

Western blot analysis was performed under standard conditions after cell lysis with an SDS sample buffer containing PhosphoSTOP (Roche, Indianapolis, IN). A 5 μg sample of each protein extract was separated by SDS-PAGE and transferred onto an Immobilon membrane (Millipore, Bedford, MA). To detect immunoreactive proteins, we used HRP-conjugated anti-mouse or anti-rabbit secondary antibodies (CST) and Pierce Western Blotting Substrate Plus (Thermo Fisher Scientific). To quantify protein levels, individual protein band images of proteins were scanned and analyzed using ImageJ software. Then, their intensities were normalized against FLAG or SARM1 as an internal control.

### 2.10 Immunostaining

Cells were incubated with 4% paraformaldehyde (PFA) for 30 min at room temperature, then permeabilized with 1% Triton X-100 in PBS for 20 min, and blocked with a buffer containing 10% skim milk in PBS-T for 30 min. Next, the cells were incubated with a rabbit FLAG antibody (MBL; cat# PM020, dilution 1:100) and a mouse Myc-tag antibody (CST; cat# 2276, dilution 1:100) overnight at 4 °C. After washing with PBS-T, the cells were stained with Alexa Fluor 488 goat anti-rabbit IgG antibody (Thermo Fisher Scientific, dilution 1:100) and Alexa Fluor 594 goat anti-mouse IgG antibody (Thermo Fisher Scientific, dilution 1:100) for 2 h at room temperature. The cells were washed again with PBS-T and mounted using VECTASHIELD Mounting Medium with DAPI (Vector Laboratories, Burlingame, CA). Finally, the samples were examined with an LSM780 confocal laser scanning microscope (Carl Zeiss, Jena, Germany).

To optically observe neurites, we performed immunostaining of NF-L and β3-tubulin in neurons. At the end of treatment, the same volume of 4% PFA to medium was added into each well of plates to fix cells for 10 min at room temperature. After removal of the solution, the cells were fixed again by 4% PFA for 20 min. After being washed with PBS, the cells were permeabilized with 0.2% TritonX-100 in PBS for 20 min, then were incubated with a blocking buffer (10% skim milk in 0.1% PBST) for 20 min. Next, the cells were incubated with a mouse anti-NF-L monoclonal antibody (Santa Cruz Biotechnology, cat# c-20012, dilution 1:200 in the blocking buffer) or a rabbit anti-β3-tubulin antibody (CST, cat# 5568, dilution 1:200 in the blocking buffer) for 18 h at 4 °C. After being washed in PBS, the cells were incubated with Alexa 594 goat anti-mouse IgG antibody (dilution 1:200 in the blocking buffer) or Alexa Fluor 594 goat anti-rabbit IgG antibody (dilution 1:200 in the blocking buffer) for 2 h at RT. After being washed in PBS, the cells were incubated with Hoechst 33342 (Thermo Fisher Scientific, dilution 1:2,000 in PBS) for 20 min, and were washed again in PBS. Finally, the samples were observed under a BZ-X700 fluorescence microscope (Keyence, Osaka, Japan). For the quantification of neurite integrity, the fluorescence intensities of NF-L, β3-tubulin, and Hoechst 33342 were scanned and analyzed using ImageJ software. Then, fluorescence intensities of NF-L and β3-tubulin were normalized against nuclear numbers.

### 2.11 Statistical analysis

Before conducting statistical analysis, each experiment was performed three times. The results are presented as means ± S. D. All statistical tests were performed using EZR (Saitama Medical Center, Jichi Medical University, Saitama, Japan) (Kanda, 2013), a graphical interface for R (The R Foundation for Statistical Computing, Vienna, Austria). One-way or Two-way ANOVA was used for comparisons. If the ANOVA indicated a significant difference, Tukey’s test was applied as a *post hoc* analysis. *p*-values less than 0.05 were considered statistically significant.

## 3 Results

### 3.1 Anticancer drug treatment enhances SARM1 ubiquitination

**Figure 1:**
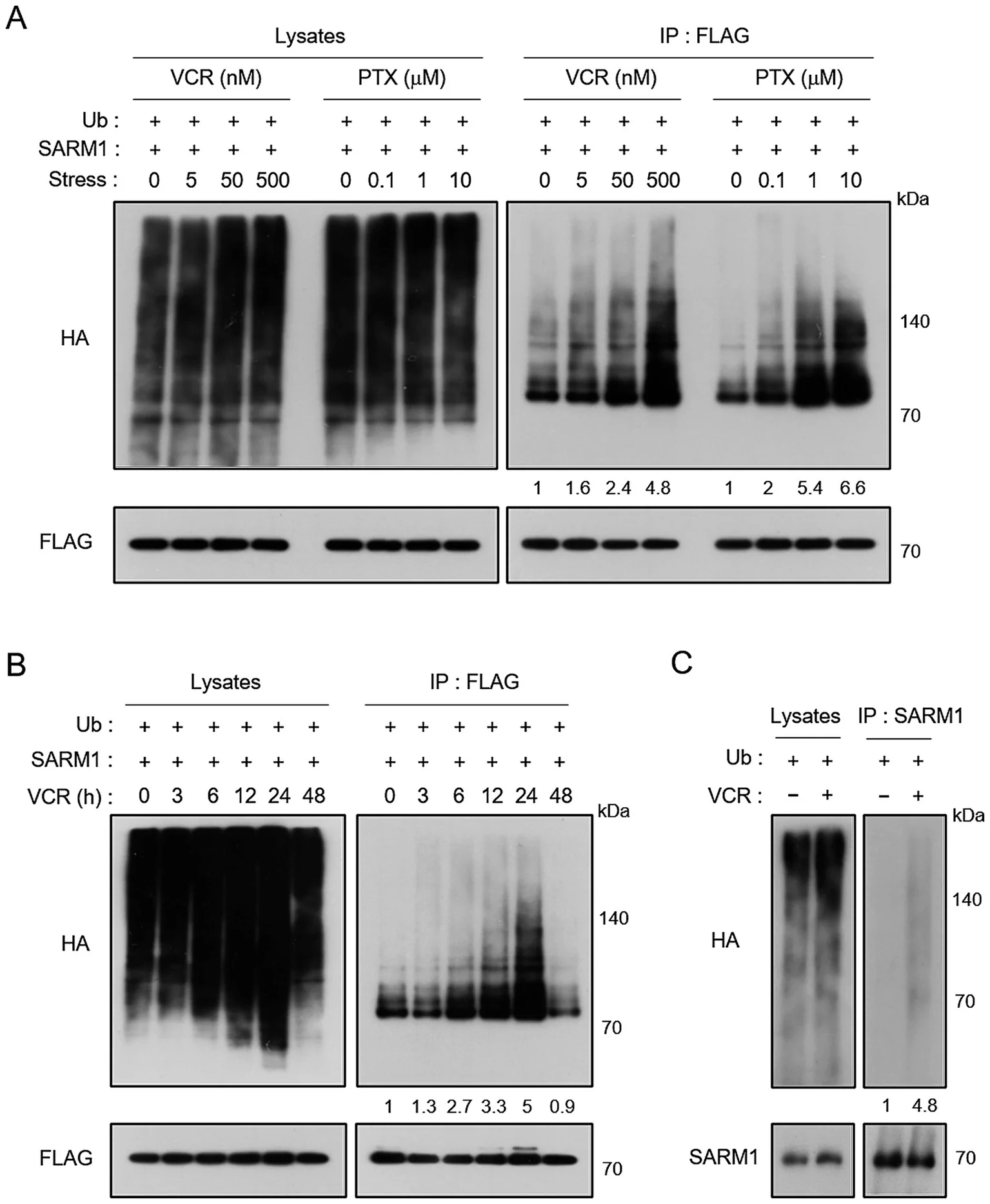
Induction of SARM1 ubiquitination under treatment of anticancer drugs. Immunoblots of transfected cell lysates (Lysates) and immunoprecipitates using the indicated antibody (IP). The ubiquitination (HA) band intensities were normalized to those of SARM1 (FLAG or SARM1), and the corrected values are shown below the panel **(A)** Dose response of SARM1 ubiquitination triggered by vincristine (VCR) and paclitaxel (PTX). HA-ubiquitin and SARM1-FLAG were overexpressed in HEK293T cells, treated with 0–500 nM VCR or 0–10 μM PTX for 24 h. **(B)** Time course of SARM1 ubiquitination. HEK293T cells overexpressing HA-ubiquitin and SARM1-FLAG were treated with 500 nM VCR for 0–48 h. **(C)** Ubiquitination of endogenous SARM1. HA-ubiquitin was overexpressed in SH-SY5Y cells, which were treated with 50 nM VCR for 24 h. Endogenous SARM1 was immunoprecipitated with an anti-SARM1 antibody.

The anticancer drugs vincristine and paclitaxel target microtubules, including axonal microtubules, leading to CIPN (Staff et al., 2017). SARM1 is a key player in axon degeneration related to CIPN, and axon degeneration can be prevented by knocking out or blocking SARM1-NAD^+^ cleavage activity (Geisler et al., 2016; Bosanac et al., 2021; Murata et al., 2025). To study changes in SARM1 during anticancer drug treatment, HEK293T cells were engineered to overexpress SARM1-FLAG and HA-ubiquitin, then treated with vincristine or paclitaxel. SARM1-FLAG was isolated through immunoprecipitation using the FLAG tag. The ubiquitination level of SARM1 increased in a dose-dependent way with vincristine or paclitaxel (Figure 1A). Vincristine-induced SARM1 ubiquitination increased over time, peaking at 24 h, then decreased after 48 h (Figure 1B). To verify ubiquitination of endogenous SARM1 in neuronal cells, SH-SY5Y cells were treated with vincristine for 24 h. As shown in Figure 1C, ubiquitination of endogenous SARM1 was also elevated after vincristine treatment. These findings suggest that SARM1 undergoes ubiquitination during activation triggered by anticancer drugs.

### 3.2 SARM1 interacts with the E3 ubiquitin ligase TRIM32

**Figure 2:**
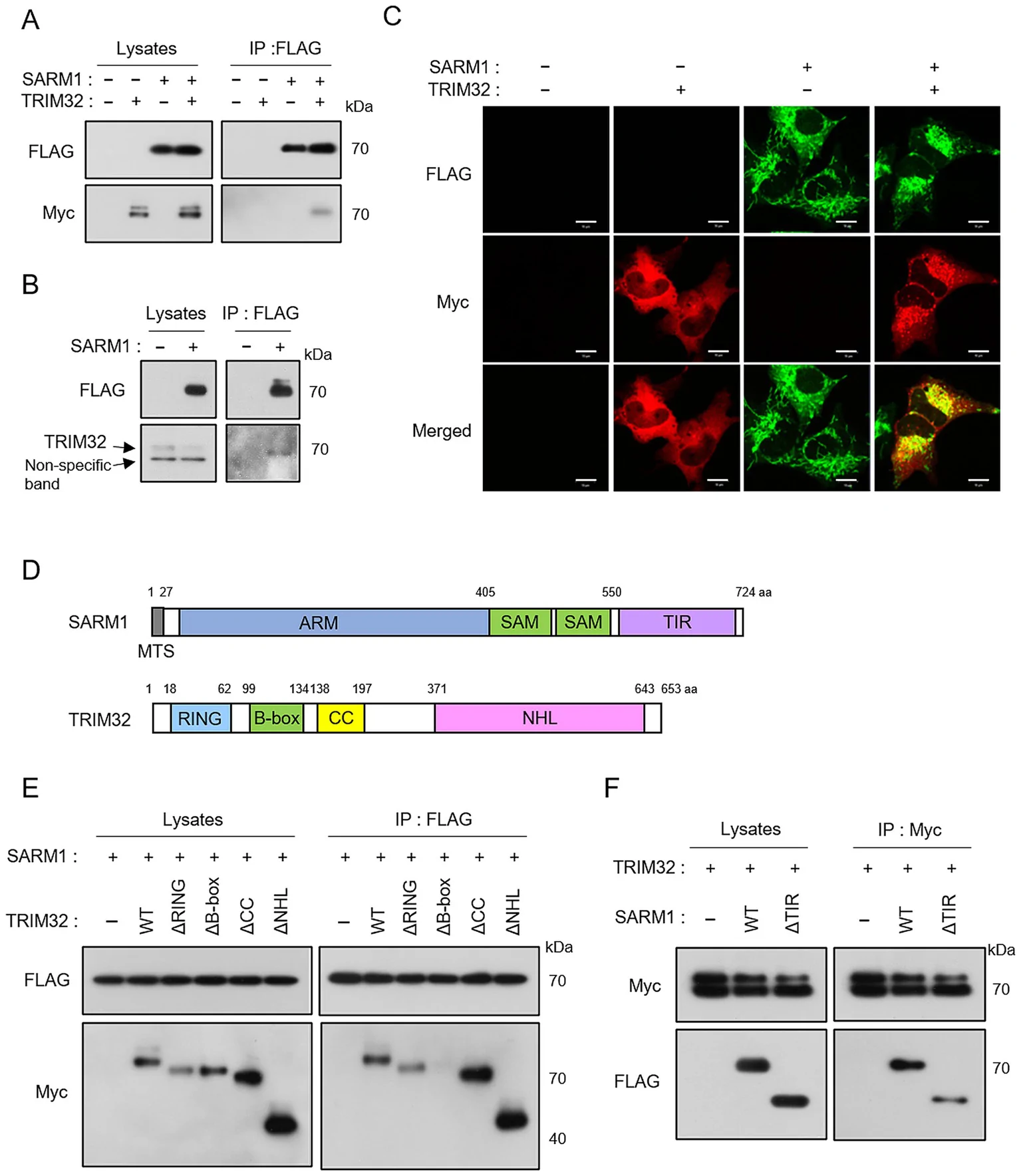
Interaction of SARM1 and TRIM32. **(A,B)** SARM1 interacts with both exogenous and endogenous TRIM32. HEK293T cells were transfected with the indicated plasmids, and SARM1-FLAG was immunoprecipitated using anti-FLAG affinity gel. **(C)** Co-localization of SARM1 and TRIM32. SARM1-FLAG and TRIM32-Myc were overexpressed in SH-SY5Y cells. Scale bar: 10 μm. **(D)** Schematic representation of human SARM1 and TRIM32. MTS: mitochondrial targeting signal; ARM: armadillo/HEAT motifs; SAM: sterile alpha motif; TIR: Toll/interleukin-1 receptor. RING: short for really interesting new gene finger; B-box: B-box-type zinc finger; CC: coiled-coil; NHL: NHL repeat. **(E)** Interaction analysis of full-length SARM1 with TRIM32 domain deletion mutants. **(F)** Interaction analysis of full-length TRIM32 with SARM1 domain deletion mutants.

**Figure 3:**
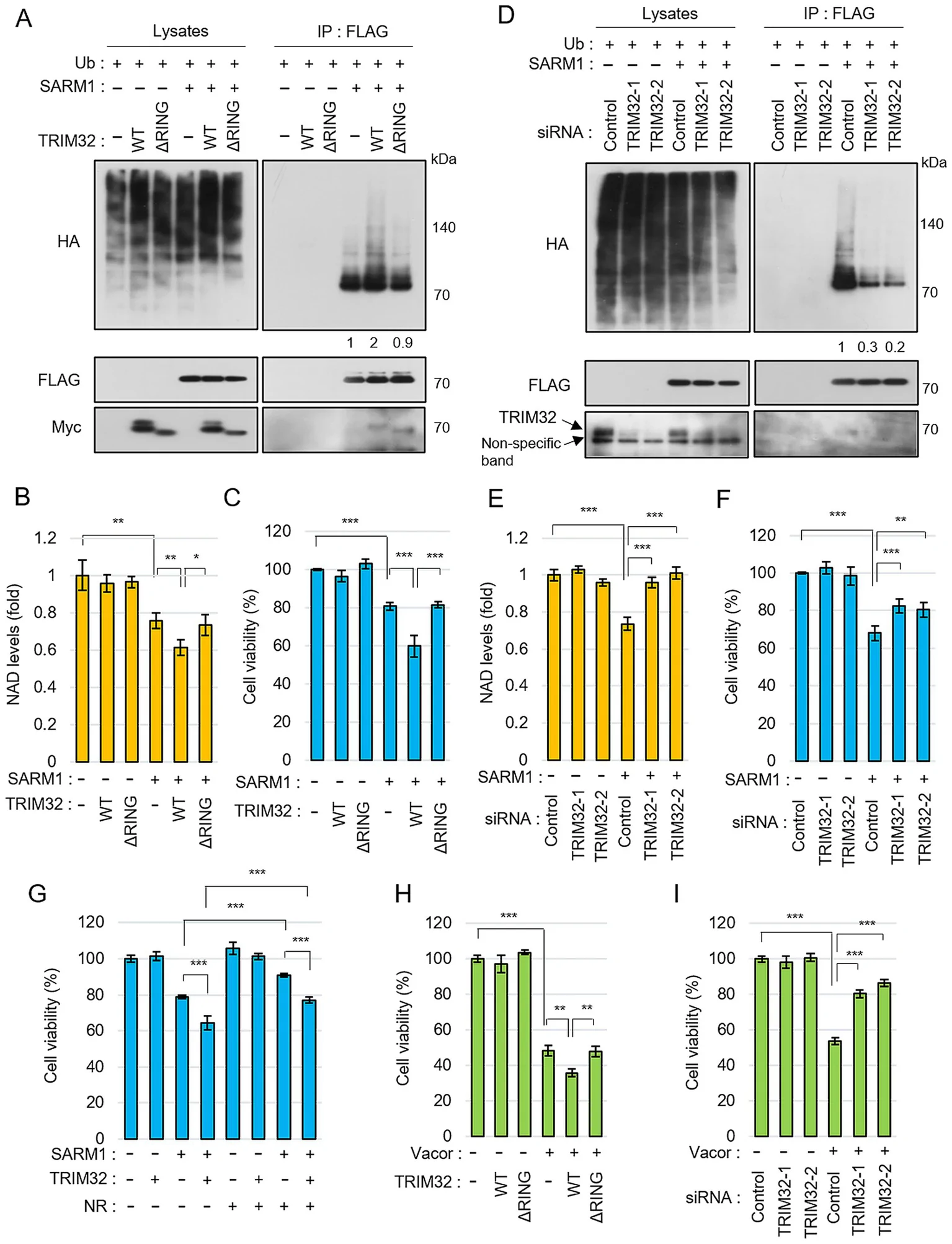
Activation of SARM1 by TRIM32-mediated ubiquitination. **(A–C)** TRIM32 promotes SARM1 ubiquitination and SARM1-induced cytotoxicity. HEK293T cells were used to overexpress SARM1-FLAG and HA-ubiquitin along with TRIM32-WT-Myc or TRIM32-ΔRING-Myc. Ubiquitination of immunoprecipitated SARM1 **(A)**, NAD^+^ levels **(B)**, and cell viability **(C)** were assessed. The ubiquitination (HA) band intensities were normalized to those of SARM1 (FLAG), and the corrected values are shown below the panel. **(D–F)** Knockdown of TRIM32 decreases SARM1 ubiquitination and SARM1-induced cytotoxicity. HEK293T cells were transfected with the specified siRNA for 24 h, then transfected with HA-ubiquitin and SARM1-FLAG for another 24 h. Ubiquitination of immunoprecipitated SARM1 **(D)**, NAD^+^ levels **(E)**, and cell viability **(F)** were measured. The ubiquitination (HA) band intensities were normalized to those of SARM1 (FLAG), and the corrected values are shown below the panel. **(G)** Suppression of SARM1-induced cell death by nicotinamide riboside (NR). HEK293T cells overexpressing SARM1-FLAG and TRIM32-Myc were treated with 1 mM NR for 24 h. **(H,I)** TRIM32 plays a role in activating endogenous SARM1. TRIM32 was overexpressed using the indicated plasmids **(H)** or downregulated with the specified siRNAs **(I)** in SH-SY5Y cells, and these transfected cells were treated with 25 μM Vacor for 8 h. *p < 0.05, **p < 0.01, ***p < 0.001.

Next, we examined the E3 ubiquitin ligase involved in SARM1 ubiquitination. Activated SARM1 localizes to mitochondria under stress conditions and decreases ATP production by inhibiting mitochondrial respiration (Murata et al., 2018; Xue et al., 2021). It is believed that ubiquitination plays a role in this activation process, as USP13 has been reported to deubiquitinate SARM1, thereby reducing its activity (Yue et al., 2023). Based on this, we investigated proteins that localize to mitochondria and can ubiquitinate. It has been reported that stimulator of interferon genes (STING), involved in antiviral responses, is negatively regulated by USP13-mediated deubiquitination (Sun et al., 2017; Lin et al., 2025) and positively regulated by TRIM32-mediated ubiquitination (Zhang et al., 2012). Therefore, we hypothesized that SARM1 might also be regulated by ubiquitination, similar to STING, and analyzed its interaction with TRIM32. SARM1-FLAG and TRIM32-Myc were overexpressed in HEK293T cells, and SARM1-FLAG was immunoprecipitated using the FLAG tag, confirming the interaction between SARM1 and TRIM32 (Figure 2A). The recruitment of TRIM32 to SARM1 was further confirmed by detecting endogenous TRIM32 with a commercially available TRIM32 antibody. Although the antibody detected two bands in the cell lysate, the SARM1-interacting TRIM32 band appeared as the upper band in the lane of the SARM1-FLAG immunoprecipitate (Figure 2B). The lower band in the cell lysate is nonspecific because it did not change after TRIM32 siRNA treatment (Figure 3D). Overexpression of SARM1 in SH-SY5Y cells resulted in mitochondrial localization, as previously reported (Figure 2C) (Murata et al., 2013). When TRIM32 was overexpressed alone, its localization was widespread throughout the cytoplasm. Co-overexpression of SARM1 and TRIM32, however, altered the localization of some TRIM32 molecules, leading to colocalization with SARM1 on mitochondria (Figure 2C).

SARM1 includes a mitochondrial targeting sequence (MTS), an autoinhibitory ARM domain, a SAM domain involved in multimerization, and a TIR domain responsible for the catalytic cleavage of NAD^+^ (Figure 2D). TRIM32 contains an enzymatic RING domain, a B-box domain involved in intermolecular interactions, a coiled-coil (CC) domain, and an NHL domain (Figure 2D). To identify which TRIM32 domain interacts with SARM1, full-length SARM1-FLAG and TRIM32-3Myc-6His mutants lacking each domain were co-overexpressed. The 3Myc-6His tag was used to enhance the sensitivity of detection of the interaction between TRIM32 mutants and SARM1. After expressing these constructs, SARM1-FLAG was immunoprecipitated using the FLAG tag. The interaction between SARM1 and TRIM32 was most significantly reduced by deletion of the B-box domain (Figure 2E), suggesting that the B-box domain of TRIM32 interacts with SARM1. Next, full-length TRIM32-Myc and SARM1-FLAG mutants lacking each domain were co-overexpressed, and TRIM32-Myc was immunoprecipitated using the Myc tag. In this analysis, the expression levels of SARM1 and TRIM32 were significantly lower when the ARM- and SAM-domain-deleted mutants were expressed compared to the wild type, making comparisons difficult. Therefore, we compared wild-type and TIR domain-deleted SARM1 and found that SARM1 and TRIM32 still interacted even without the TIR domain (Figure 2F). These results suggest that the TIR domain of SARM1 does not contribute to the SARM1-TRIM32 interaction.

### 3.3 TRIM32 activates SARM1 via ubiquitination

To confirm that TRIM32 ubiquitinates SARM1, wild-type (WT) or a TRIM32-Myc mutant lacking the RING domain (ΔRING) was co-overexpressed with SARM1-FLAG and HA-ubiquitin. TRIM32-WT promoted SARM1 ubiquitination, while deletion of the RING domain abolished this effect (Figure 3A). Furthermore, TRIM32-WT enhanced the SARM1-induced decrease in NAD^+^ levels and cell viability, but deletion of the RING domain eliminated this effect (Figures 3B,C). To analyze the effect of endogenous TRIM32 on SARM1 ubiquitination and activity, TRIM32 was knocked down using siRNA. Two types of siRNA against TRIM32 reduced TRIM32 expression (Figure 3D, the upper band of TRIM32), and TRIM32 knockdown decreased the ubiquitination level of SARM1 (Figure 3D). Knockdown of TRIM32 also suppressed the SARM1-induced decrease in NAD^+^ levels and cell viability (Figures 3E,F). Because supplementation with the NAD^+^ precursor nicotinamide riboside (NR) suppressed the SARM1-induced reduction in cell viability (Figure 3G), this phenomenon is considered NAD^+^-dependent. To study the effect of TRIM32 on endogenous SARM1 activation, SH-SY5Y cells were manipulated to overexpress or downregulate TRIM32 and then treated with Vacor. Vacor is a neurotoxin that is converted inside the cell into a structure similar to NMN, which activates SARM1 (Loreto et al., 2021). TRIM32-WT increased Vacor-induced cell weakness, while deletion of the RING domain prevented this (Figure 3H). Knocking down TRIM32 significantly reduced the loss of cell viability caused by Vacor (Figure 3I). These findings suggest that TRIM32-mediated ubiquitination helps activate SARM1.

### 3.4 TRIM32 modifies Lys173 and Lys375 of SARM1 with Lys27-linked ubiquitin chains

**Figure 4:**
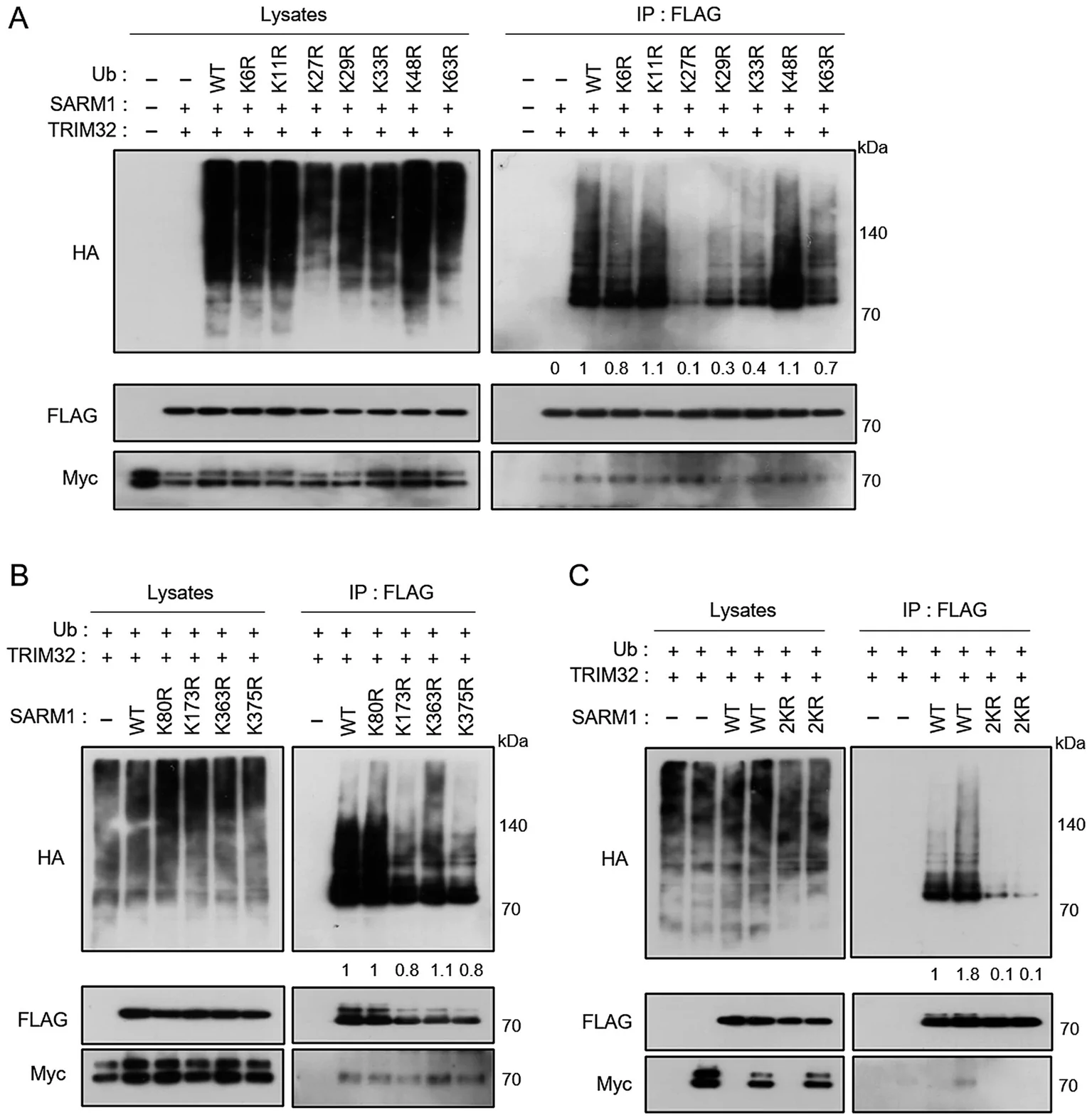
Identification of ubiquitin chain types and ubiquitination sites modified by TRIM32 in SARM1. **(A–C)** HEK293T cells were transfected with the indicated plasmids for 24 h, and SARM1-FLAG was immunoprecipitated with anti-FLAG affinity gel. **(A)** Lys27 ubiquitin mutations decreased SARM1 ubiquitination. **(B)** Mutations at Lys173 or Lys375 partially reduced SARM1 ubiquitination. **(C)** Double mutations at Lys173 and Lys375 (2KR) in SARM1 markedly reduced SARM1 ubiquitination. The ubiquitination (HA) band intensities were normalized to those of SARM1 (FLAG), and the corrected values are shown below the panel.

The ubiquitin chains attached to substrate proteins through ubiquitination enzymes vary in structure depending on the target lysine residues. These differences influence various protein fates and directly affect multiple cellular processes (Komander and Rape, 2012). To determine the type of ubiquitin chain attached to SARM1 by TRIM32, wild-type and seven different HA-ubiquitin variants with Arg substitutions (WT, K6R, K11R, K27R, K29R, K33R, K48R, and K63R) were overexpressed alongside SARM1-FLAG and TRIM32-Myc. Ubiquitination of SARM1 mediated by TRIM32 was most notably reduced when Lys27 was replaced by Arg, indicating that TRIM32 attaches Lys27-linked ubiquitin chains to SARM1 (Figure 4A). Next, we aimed to identify the ubiquitination sites on SARM1. Based on the post-translational modification database (Udeshi et al., 2013), four lysines (Lys80, Lys173, Lys363, and Lys375) in SARM1 were identified using mass spectrometry, and mutants (K80R, K173R, K363R, and K375R) were created. Ubiquitination of SARM1 by TRIM32 was reduced when Lys173 and Lys375 were replaced with Arg, although not significantly (Figure 4B). Therefore, a double-mutant vector, SARM1-K173R/K375R (2KR), with both lysines replaced by Arg, was developed. The TRIM32-mediated ubiquitination of SARM1 was significantly decreased with these double mutations (Figure 4C). Additionally, co-overexpression of TRIM32 with SARM1-2KR did not increase the NAD^+^-cleavage activity or cytotoxicity of SARM1 (Supplementary Figures S1A,B). These results suggest that TRIM32 modifies Lys173 and Lys375 of SARM1 with Lys27-linked ubiquitin chains, and that this ubiquitination helps alter SARM1’s structure and activate it.

### 3.5 Regulation of post-translational modification of SARM1

**Figure 5:**
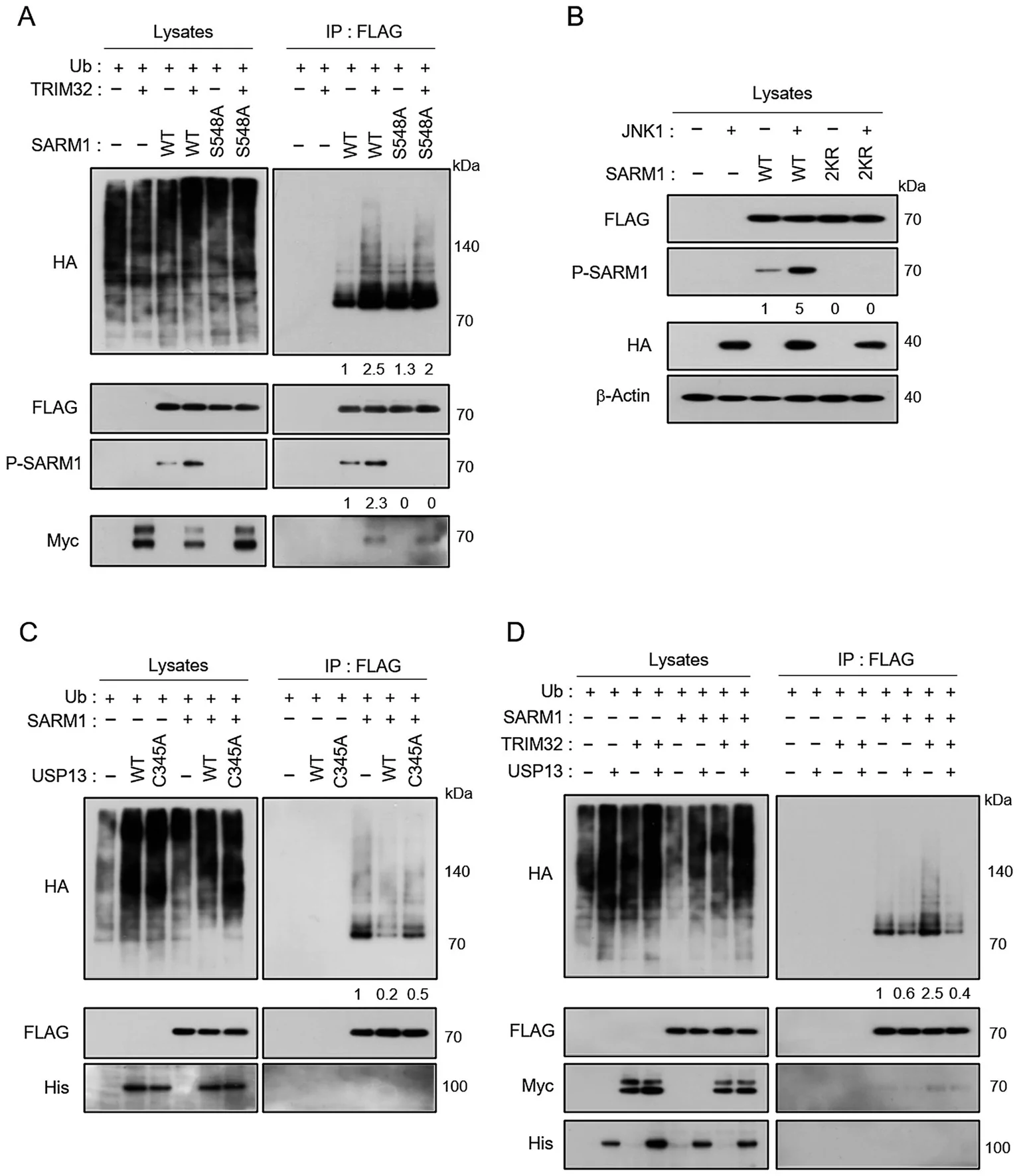
Relationship between SARM1 ubiquitination and other post-translational modifications. **(A–D)** HEK293T cells were transfected with the indicated plasmids for 24 h, and SARM1-FLAG was immunoprecipitated with anti-FLAG affinity gel **(A,C,D)**. **(A)** Ubiquitination and phosphorylation of SARM1 wild type (WT) and SARM1-S548A mutant by TRIM32. **(B)** Phosphorylation of SARM1-WT and SARM1-2KR mutant by JNK1. **(C)** Deubiquitination of SARM1 by USP13-WT or USP-C345A mutant. **(D)** Removal of TRIM32-mediated ubiquitination of SARM1 by USP13. The band intensities for ubiquitination (HA) and phosphorylation (P-SARM1) were normalized to those of SARM1 (FLAG), and the corrected values are shown below the panel.

We previously reported that JNK phosphorylates SARM1 at Ser548, contributing to its activation (Murata et al., 2018). To clarify the relationship between Ser548 phosphorylation and ubiquitination at Lys173 and Lys375, we examined SARM1-S548A and SARM1-2KR mutants. Initially, SARM1-FLAG (either wild-type or S548A mutant) was co-expressed with HA-ubiquitin and TRIM32-Myc, then analyzed for changes in ubiquitination and phosphorylation. Ubiquitination of SARM1 increased with co-overexpression of TRIM32 in both wild-type and S548A (Figure 5A). Phosphorylation of SARM1 was detected using an antibody specific for Ser548 phosphorylation; no band appeared with S548A mutant (Figure 5A). Co-overexpression of TRIM32 also increased the phosphorylation level of SARM1-WT (Figure 5A). Next, SARM1-WT-FLAG or SARM1-2KR-FLAG was co-overexpressed with HA-JNK1, and changes in phosphorylation were assessed. The foreign SARM1-WT was faintly phosphorylated in the absence of JNK1 co-expression, and its basal level was further increased by JNK1 overexpression (Figure 5B). Notably, the baseline phosphorylation seen in the wild type was eliminated in SARM1-2KR, and even co-overexpression of JNK1 did not increase phosphorylation (Figure 5B). Regarding these results, SARM1-induced reduction in NAD^+^ levels and cytotoxicity were decreased in S548A, whereas co-expression of TRIM32 reduced NAD^+^ levels and cell viability similarly to that in the wild-type (Supplementary Figures S1C,D). These findings suggest that TRIM32 catalyzes SARM1 ubiquitination, followed by JNK-mediated phosphorylation. Third, we examined USP13, which has been reported to deubiquitinate SARM1 (Yue et al., 2023). To confirm that USP13’s enzymatic activity is necessary for SARM1 deubiquitination, we compared wild-type USP13 with a USP13 mutant (C345A). Cys345 in USP13 is important for its deubiquitination activity (Lin et al., 2025). Under our experimental conditions, similar to those used for the TRIM32-SARM1 co-immunoprecipitation, no appreciable interaction between SARM1 and USP13 was observed. However, USP13-WT reduced SARM1 ubiquitination, and its deubiquitination was suppressed by the C345A mutation in USP13 (Figure 5C), suggesting a very weak catalytic interaction between them. Additionally, USP13-WT lowered SARM1 ubiquitination levels, which were increased by TRIM32-WT (Figure 5D). These results suggest that TRIM32 and USP13 reciprocally regulate SARM1 ubiquitination at the same target sites, a process critical for subsequent JNK-mediated phosphorylation and catalytic activation of SARM1.

### 3.6 TRIM32 promotes neurite degeneration and cell death

**Figure 6:**
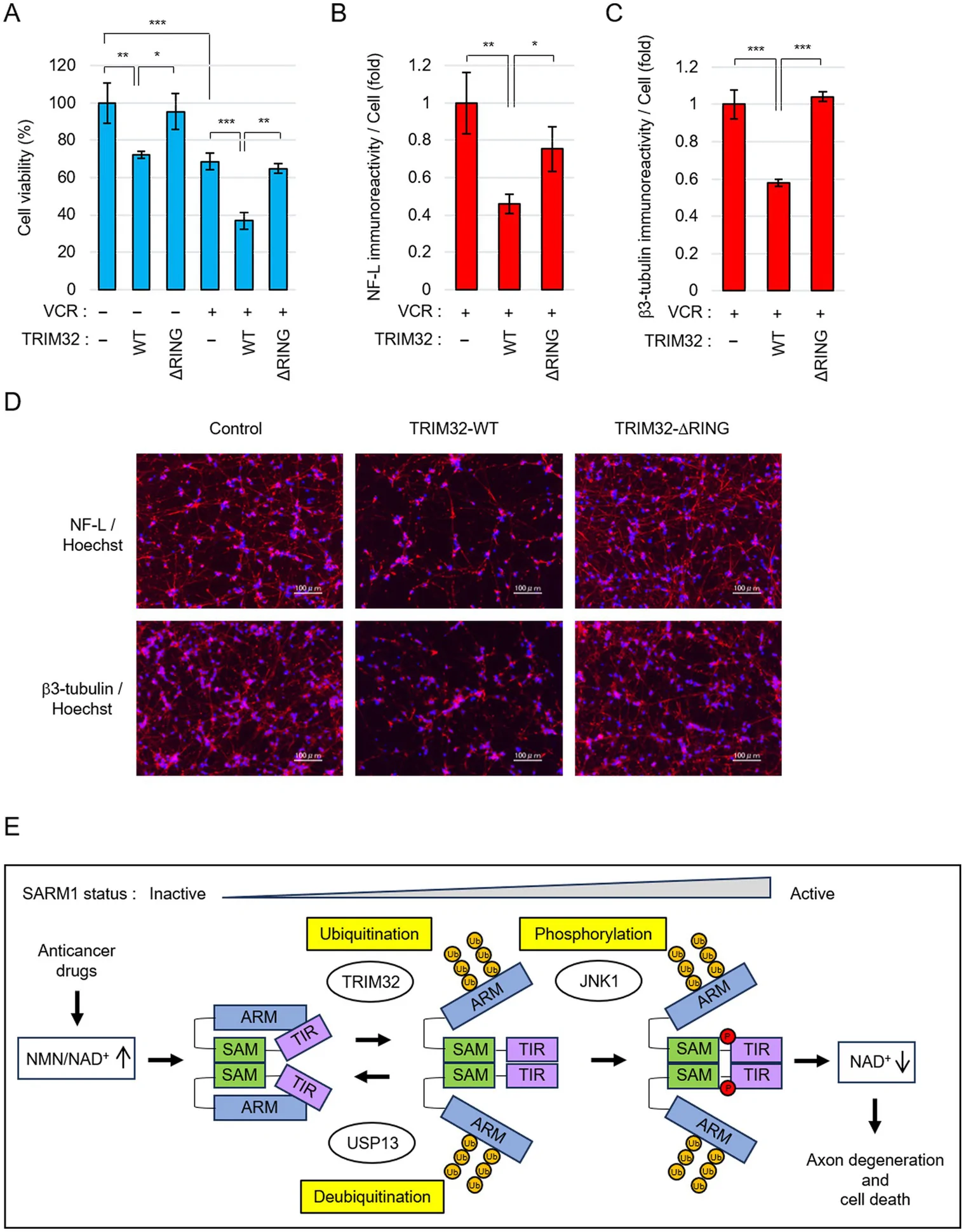
TRIM32 is involved in neurite degeneration and cell death. **(A–D)** Human iPSC-derived neurons co-expressing a puromycin resistance gene and TRIM32-WT, TRIM32-ΔRING, or a control vector were treated with 50 nM vincristine (VCR) for 18 h. **(A)** Cell viability. **(B)** Quantification of NF-L immunoreactivity. **(C)** Quantification of β3-tubulin immunoreactivity. **(D)** Representative images of cell morphologies, scale bar: 100 μm. **(E)** A working model for the regulation of SARM1 activity by post-translational modification. *p < 0.05, **p < 0.01, ***p < 0.001.

Finally, we investigated whether TRIM32 has a role in axon degeneration and cell death using human iPS cell-derived neurons. Stable neural stem cell lines co-expressing TRIM32-WT or TRIM32-ΔRING along with a puromycin resistance gene were created, then differentiated into neurons. Compared to control neurons, TRIM32-WT increased cell death, whereas TRIM32-ΔRING did not cause this effect (Figure 6A). The cell death induced by TRIM32-WT were further intensified by vincristine (VCR) treatment (Figure 6A). Morphological observation showed that TRIM32-WT increased VCR induced neurite degeneration, whereas TRIM32-ΔRING did not cause this effect (Figures 6B–D). These results suggest that TRIM32 negatively affects neuronal survival.

## 4 Discussion

In this study, we identified TRIM32 as an E3 ubiquitin ligase for SARM1 and elucidated the mechanism of SARM1 activation via ubiquitination and phosphorylation (Figure 6E). TRIM32 attaches Lys27-linked ubiquitin chains to Lys173 and Lys375 on SARM1, inducing structural changes that activate the protein. After ubiquitination, JNK1 further activates SARM1 by phosphorylating Ser548, while USP13 removes TRIM32-mediated ubiquitination. These findings significantly impact the field of neurological diseases, as TRIM32 is expressed in neurons and has been linked to mitochondrial dysfunction, cell death, and the progression of Parkinson’s disease (Goyani et al., 2024).

Goyani et al. showed that TRIM32 accumulates at mitochondria under oxidative stress, leading to cell death by altering levels of X-linked inhibitor of apoptosis (XIAP) (Goyani et al., 2024). In line with this, we also confirmed that TRIM32 co-localizes and interacts with SARM1 on mitochondria (Figure 2). SARM1 causes mitochondrial dysfunction and cell death by depleting NAD^+^. These results suggest that TRIM32 impairs neuronal survival by affecting various mitochondrial substrates through mitochondrial translocation. Another known mitochondrial target of TRIM32 is STING, involved in antiviral responses. STING acts as an adaptor during viral infections, activating IRF3 and NF-κB, and inducing IFN-*β* production. A report suggests that TRIM32 ubiquitinates STING, promoting its interaction with TBK1 and activating IRF3 and NF-κB (Zhang et al., 2012). Meanwhile, USP13 can suppress antiviral responses by deubiquitinating STING, reducing its interaction with TBK1 (Sun et al., 2017). It is also shown that USP13’s removal of ubiquitin from STING leads to its degradation via autophagy (Lin et al., 2025). Therefore, ubiquitination of STING by TRIM32 enhances signal transduction and stabilizes the protein, unlike proteasomal degradation via Lys48-linked ubiquitination. The ubiquitination of SARM1 by TRIM32 involves Lys27-linked chains (Figure 4), which aid in activating SARM1 when attached to the ARM domain. Similar to STING, SARM1 resides in mitochondria and may be regulated by TRIM32 and USP13 via similar pathways in stressed neuronal cells.

SARM1 plays a crucial role not only in neuronal cells but also in immune cells, as it can regulate mitochondria and the inflammasome, thereby associating with pyroptosis and the release of inflammatory cytokines (Carty et al., 2019; Sugisawa et al., 2024). SARM1 activation induces mitochondrial damage and cell death, thereby eliminating hyperactivated immune cells via pyroptosis. In contrast to promoting pyroptosis, SARM1 directly binds to NLRP3, attenuates its function, and reduces the activation of inflammatory cytokines (Carty et al., 2019; Oliveira et al., 2025). Reduced SARM1 expression in macrophages has been reported to enhance the induction of inflammatory cytokines and increases the severity of inflammatory bowel disease (Fan et al., 2024). Given that the NAD^+^-cleavage activity of SARM1 is significantly altered by regulation via TRIM32 and USP13, it would be of great interest to investigate how the regulation of SARM1 ubiquitination regulation affects the immune system.

Previously, we demonstrated that SARM1 is activated by JNK-dependent phosphorylation (Murata et al., 2018; Murata et al., 2023), and here we provide a more detailed understanding of its relationship to ubiquitination (Figure 5). Ubiquitination by TRIM32 increased SARM1 phosphorylation, while blocking ubiquitination decreased it. This indicates that under stress conditions, such as exposure to anticancer drugs, TRIM32 attaches ubiquitin to Lys173 and Lys375 within the suppressor ARM domain of SARM1, weakening its interaction with the catalytic TIR domain. This process likely involves NMN binding to the ARM domain, causing SARM1 to adopt a more flexible, open shape. This change in shape makes it easier for JNK1 to phosphorylate SARM1 at Ser548, which may help stabilize its active form in a persistent manner (Figure 6E). In addition to our earlier finding that JNK1 phosphorylates SARM1, we also highlighted the importance of SARM1 ubiquitination by TRIM32 versus USP13. Although JNK-mediated phosphorylation of SARM1 is negatively regulated by the STAT1/3 pathway (Murata et al., 2023), the upstream pathway regulating TRIM32 remains unclear; we plan to investigate this further. Interestingly, other modifications like deacetylation by SIRT3 and cleavage by caspase-3 have also recently been shown to regulate SARM1. Chen et al. (2026) demonstrated that, in a diabetic nerve damage model, SIRT3 removes an acetyl group from Lys641 in SARM1’s TIR domain, increasing its activity to degrade NAD^+^. Conversely, Shi et al. (2026) found that during cell death, caspase-3 cleaves the ARM domain of SARM1, removing autoinhibition and enhancing its activity. Thus, SARM1 activation is clearly regulated by multiple post-translational modifications controlled by various pathways. Understanding these processes could aid in developing treatments that protect neural pathways.

Finally, a limitation of the current study is that the *in vivo* role of TRIM32-mediated regulation of SARM1 ubiquitination remains unclear. In neurological diseases, SARM1 is thought to contribute to pathogenesis not only by promoting axonal degeneration but also through its association with neuroinflammation; indeed, the phenotype of SARM1-dependent neuropathy is inhibited by macrophage depletion (Dingwall et al., 2022). Furthermore, SARM1 deficiency in experimental models of Alzheimer’s disease has been shown to reduce β-amyloid protein deposition and TNF-*α* signaling, thereby offering protection against neuroinflammation (Miao et al., 2024). Future research using *in vivo* models of CIPN and other neurological diseases is expected to further elucidate the importance of SARM1 post-translational modifications in axonal degeneration and neuroinflammation, potentially leading to the establishment of novel preventive and therapeutic strategies.

## Data Availability

The raw data supporting the conclusions of this article will be made available by the authors, without undue reservation.
