# Supplementary material for: New mechanistic insight into SARM1 activation: TRIM32-mediated ubiquitination is a key lever that actuates SARM1’s catalytic action, leading to axon degeneration and cell death
Source: Front Neural Circuits. 2026 Aug 20;20:1842408. doi: 10.3389/fncir.2026.1842408 (PMC13538860; doi:10.3389/fncir.2026.1842408)
Supplement: Supplementary file 1 [file Data_Sheet_1.PDF]

Supporting Information for

New mechanistic insight into SARM1 activation: TRIM32-mediated ubiquitination is a key lever that actuates SARM1's catalytic action, leading to axon degeneration and cell death.

Hitoshi Murata*, Nahoko Tomonobu, Ken-ichi Yamamoto, Rie Kinoshita, Masakiyo Sakaguchi

Correspondence should be addressed to Hitoshi Murata: murata@md.okayama-u.ac.jp

This file includes:

Fig. S1

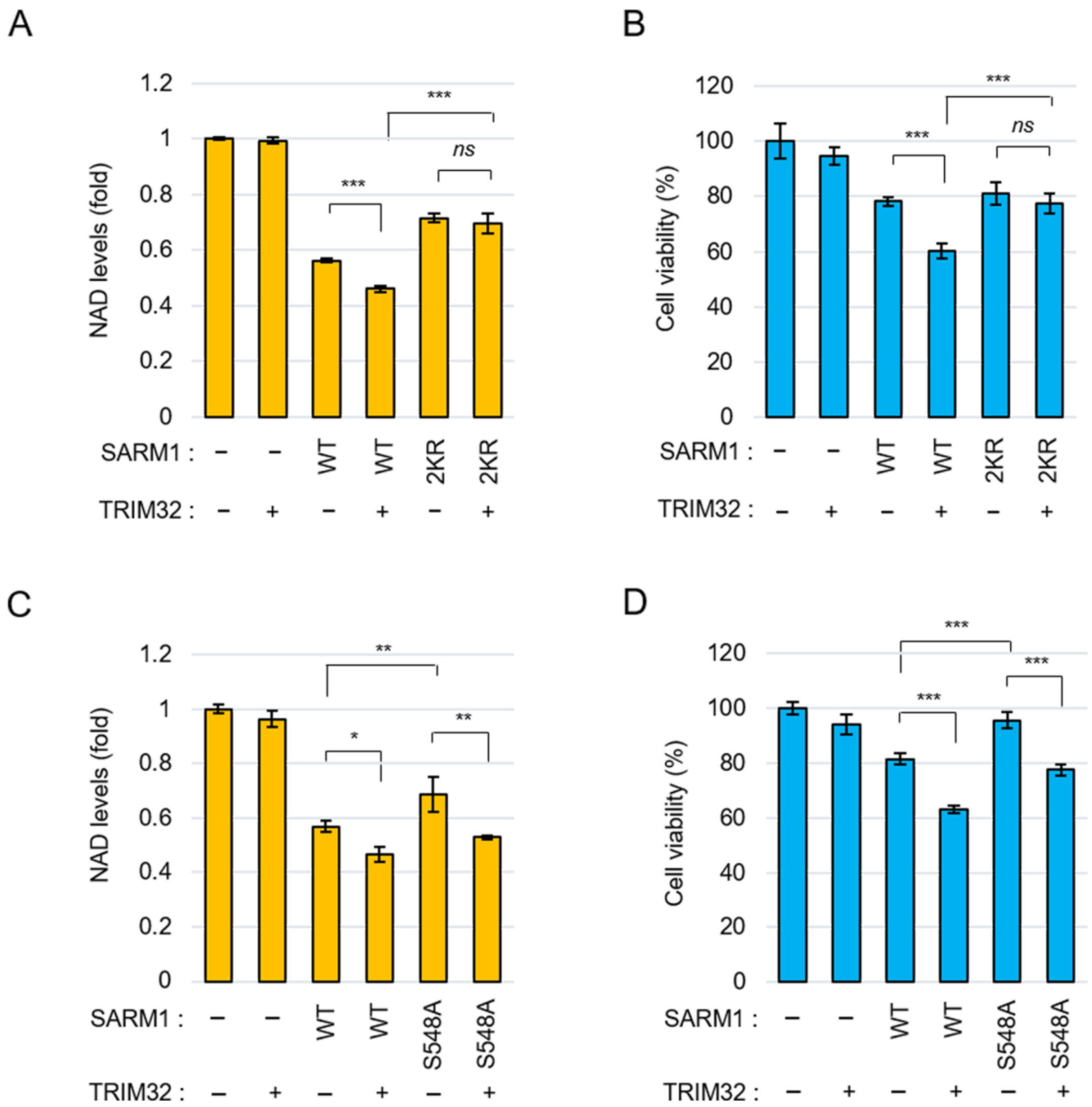

Figure S1. Regulation of SARM1 activity by TRIM32.

(A-D) HEK293T cells were transfected with the indicated plasmids for 24 hours. (A) NAD^+ levels of cells overexpressing SARM1-WT or SARM1-2KR with TRIM32. (B) SARM1-induced cytotoxicity is not increased by TRIM32 in the SARM1-2KR mutant. (C) NAD^+ levels of cells overexpressing SARM1-WT or SARM1-S548A with TRIM32. (D) SARM1-induced cytotoxicity was decreased in S548A, but co-expression of TRIM32 reduced cell viability similarly to that of wild-type. *ns*: not significant, *p<0.05, **p<0.01, ***p<0.001.
